# Feasibility and Confidence in Carotid Spectral Doppler for Pulse Detection: Training Outcomes Among Emergency Medicine Residents

**DOI:** 10.7759/cureus.85354

**Published:** 2025-06-04

**Authors:** Jeremy Carter, Connor Babbush, Ian Coe, Stephen Haight, Javier Andrade, Josie Acuña, Srikar Adhikari

**Affiliations:** 1 Emergency Medicine, University of Texas Medical Branch, Galveston, USA; 2 Emergency Medicine, University of Arizona, Tucson, USA; 3 Internal Medicine, University of Arizona, Tucson, USA; 4 Emergency Medicine, University of California, San Francisco, Fresno, Fresno, USA; 5 Emergency Medicine, Abrazo Health, Phoenix, USA

**Keywords:** bedside ultrasound, cardiac arrest, carotid doppler, medical education, point-of-care-ultrasound, return of spontaneous circulation (rosc)

## Abstract

Introduction

Carotid ultrasound has demonstrated utility in identifying the presence of a pulse, particularly in cardiac arrest. Despite this evidence, there is currently no existing literature that focuses on the training of physicians in this modality for the purpose of pulse detection. The primary objective of this study is to demonstrate that novice ultrasound users with varying levels of training can be taught a simple and repeatable method for identifying flow in the common carotid artery (CCA) using a combination of B-mode ultrasound and spectral Doppler. The secondary objective is to evaluate the confidence of emergency medicine (EM) residents with varying levels of ultrasound experience in accurately identifying the carotid pulse using spectral Doppler.

Methods

This is a cross-sectional study, using a convenience sample of emergency medicine (EM) residents. Residents with varying levels of ultrasound experience received training on obtaining CCA ultrasound images and spectral Doppler. Subjects were then asked to image the common carotid artery and obtain a spectral Doppler waveform on a standardized patient. Expert sonologists observed participants’ imaging in real time and recorded their performance. Participants completed a post-questionnaire detailing the number of previous ultrasound examinations they had performed and their comfort level with the carotid ultrasound technique.

Results

Thirty-eight residents participated in the study. The residents were able to obtain a spectral Doppler waveform from the CCA in 38 (100%) of cases, and 35 (92%) residents were able to perform this task without any assistance from the instructor. The average time to obtain a waveform was 23 seconds. Twelve (32%) residents were able to obtain a pulse within 15 seconds. After the training session, 37 (97%) residents felt comfortable identifying arterial flow in the CCA using spectral Doppler and would feel comfortable using this technique on an actual patient receiving cardiopulmonary resuscitation (CPR).

Conclusion

Spectral Doppler of the CCA may be a reasonable alternative for identifying return of spontaneous circulation (ROSC) during pulse check of patients receiving CPR. All residents were able to identify pulses using spectral Doppler, following a brief lecture, instructional video, and hands-on training session.

## Introduction

Goal-directed interventions using measures of cerebral blood flow to guide resuscitation have great potential for improving cardiopulmonary resuscitation (CPR) outcomes. Patient evaluation during cardiac arrest remains a challenge, posing a significant obstacle to favorable outcomes following CPR. Accurately determining return of spontaneous circulation (ROSC) is imperative in dictating the course of an ongoing resuscitation. Inaccurate pulse checks, more specifically, the inability to detect very faint pulses through manual palpation, may erroneously influence the determination of who receives additional interventions and who does not. Manual palpation of pulses has been shown to be inaccurate and time-consuming, especially in instances when no pulse is identified [1-3]. Despite a large body of evidence revealing the inadequacies of manual palpation of pulses during cardiac arrest, this method is still commonplace, presumptively due to a lack of training and confidence in using other methods.

When evaluating the utility and feasibility of pulse checks using different ultrasound methods during cardiac arrest, the focus should be on minimizing disruption to ongoing resuscitation efforts while ensuring prompt and accurate determination of the presence or absence of a pulse. Cardiac ultrasound performed during pulse check can provide valuable information [4,5]. However, the acquisition of images can be challenging, and cardiac ultrasound has demonstrated a potential for prolonging pulse checks, thereby interfering with the efficiency of resuscitation [6,7]. Quantitative end-tidal carbon dioxide waveform capnography can demonstrate adequate CPR and identify ROSC, but is also subject to limitations such as equipment malfunctions, delays in reading, and low tidal volumes [8,9]. More recently, transesophageal echocardiography (TEE) has been shown to provide valuable information during cardiac arrest without delaying chest compressions but is not feasible to use in all patients [10-13] Additionally, implementing TEE into resuscitation requires expensive, specialized equipment and extensive training and is not currently feasible in most emergency departments.

The common carotid artery (CCA) is an ideal location for direct non-invasive measurement of blood flow without interrupting chest compressions during CPR. Carotid ultrasound can be used for pulse checks and to help optimize chest compressions. The utility of spectral Doppler in identifying the presence or lack of pulse in peripheral arteries has been demonstrated by prior studies. A case series demonstrated the accurate identification of the presence or absence of pulse by grayscale compression sonography of the CCA and common femoral arteries [14]. Several studies have shown that carotid imaging and Doppler are reliable techniques for identifying ROSC [15,16]. With the feasibility and accuracy of CCA Doppler being demonstrated, its role as a tool aimed at optimizing the efficacy and efficiency of resuscitation efforts during cardiac arrest is reliant upon proper training. At present, there is no existing literature that focuses on the training of physicians to use CCA Doppler for the purpose of pulse detection. Integrating CCA Doppler training with pre-existing ultrasound education present at many emergency medicine (EM) residency programs has considerable potential in setting a new precedent for pulse checks among new generations of physicians during the management of cardiac arrest. The primary objective of this study is to evaluate the ability of EM resident physicians to learn and accurately obtain a spectral Doppler waveform from the CCA. The secondary objective is to evaluate the confidence of EM residents with varying levels of ultrasound experience in accurately identifying the carotid pulse using spectral Doppler.

## Materials and methods

This was a cross-sectional study conducted at two academic medical centers with two EM residency programs and a combined EM/pediatrics residency program. The emergency department (ED) at the medical centers has ongoing emergency ultrasound training programs. Hospital credentialing for point-of-care (POC) ultrasound is available for emergency physicians, following the guidelines set by the American College of Emergency Physicians (ACEP) regarding POC ultrasound usage [17]. This study was approved by the Institutional Review Board of the University of Arizona (approval number: 1806663505). The study participants were EM resident physicians from various stages of their training who voluntarily chose to participate. All residents from the three residency training programs were invited to participate, and consent was obtained from all participants. There were no exclusion criteria other than a resident choosing not to participate.

Seven weeks prior to the hands-on training day, all residents underwent a one-hour lecture focusing on the background and technique for spectral Doppler of the CCA. Two days prior to the hands-on training session, all EM residents were provided with a video link to an educational video that demonstrated the technique. The hands-on training session commenced with a 15-minute lecture and demonstration, which served to review the technique. Subsequently, each participant was asked to identify the CCA using a standardized patient. They were instructed to first locate it using B-mode imaging and then proceed to identify it using spectral Doppler techniques. Images were obtained by each participant on a standardized patient using Zonare (Zonare Medical Systems, Mountain View, CA) with a 10-5 MHz broadband linear transducer. The time to obtain the spectral waveform was collected, and each resident’s ability to obtain quality images without assistance was evaluated. The time measurement was initiated once the participant picked up the ultrasound transducer and was completed once an adequate image and waveform were obtained. An image was deemed to be of adequate quality once it was determined to be interpretable and was at the discretion of the evaluators. The evaluations were completed by EM ultrasound fellowship-trained physicians. Following the assessments, all participants completed a questionnaire detailing the number of previous ultrasound examinations they had performed, their comfort level with the technique, and their use of carotid ultrasound for pulse check during CPR. Descriptive statistics were used to summarize the data. Continuous data are presented as means, and categorical data are presented as frequencies and percentages.

## Results

A total of 38 emergency medicine residents of various post-graduate years (PGY) participated in the study (10 PGY1s, 14 PGY2s, 10 PGY3s, one PGY4, and three PGY5s). The mean total number of ultrasound examinations done by each participant prior to the study was 199 (SD + 101). The mean total number of vascular ultrasound examinations done by each participant prior to the study was 45 (SD + 39). Prior to this study, 25 (66%) residents reported receiving some training in using spectral Doppler, and 19 (50%) residents stated that they were familiar with the concept of using carotid artery ultrasound during CPR.

Basic carotid ultrasound performance metrics were recorded and summarized in Table 1. All 38 (100%) participants were able to appropriately position the probe and obtain an optimal view of the carotid artery using B-mode imaging. Thirty-five (92%) residents were able to obtain a carotid view and appropriately use the spectral Doppler without any assistance. The average time to obtain the optimal carotid artery image and Doppler signal was 23 seconds (SD + 16 seconds).

**Table 1:** Carotid ultrasound performance metrics by residents

Assessment metric	Result (number (%))
Participants obtaining optimal B-mode carotid view	38 (100%)
Participants obtaining a carotid view and using spectral Doppler without assistance	35 (92%)
Participants obtaining a Doppler signal in ≤10 seconds	7 (18%)
Participants obtaining a Doppler signal in ≤15 seconds	13 (34%)
Participants obtaining a Doppler signal in ≤20 seconds	22 (58%)

On a scale of 1 to 5 (with 1 being easy and 5 being difficult), the observer rated the participant’s difficulty in obtaining a carotid artery view at 1.2 (SD + 0.71). The observer rated the participants’ difficulty in using spectral Doppler at 1.3 (SD + 0.589) on a scale of 1 to 5, where 1 indicates the image could be easily obtained and 5 indicates significant difficulty in obtaining the image. The overall assessment of the participants’ scanning difficulty was rated at 1.2 (SD + 0.413) on the same scale. The mean image quality was rated at 4.6 (SD+0.545) on a scale of 1 to 5, where 1 indicates poor image quality and 5 indicates excellent image quality. The instructors felt that the participants easily obtained the carotid artery view and easily used the spectral Doppler 95% of the time. The observer rated the participants’ difficulty in using spectral Doppler at 1.3 (SD = 0.589) on a scale of 1 to 5, where 1 indicates the image could be easily obtained and 5 indicates significant difficulty in obtaining the image. The overall assessment of the participants’ scanning difficulty was rated at 1.2 (SD = 0.413) on the same scale.

After the session, 36 (95%) participants answered that they felt comfortable using spectral Doppler, and 24 (63%) felt that using this technique during CPR would not be challenging. On a scale of 1 to 10, with 1 indicating low confidence and 10 indicating high confidence, participants reported a confidence level of 8.5 (SD = 1.2) in using spectral Doppler. Participants indicated that performing a carotid ultrasound for CPR purposes will be “moderately challenging,” with an average difficulty rating of 6.7 (SD = 2.3) on a scale of 1 to 10, where 1 represents “not challenging” and 10 represents “extremely challenging.” All residents agreed that carotid ultrasound can be used to accurately assess for a pulse, during the pulse, check of a patient receiving CPR. After the training session, 37 (97%) residents felt comfortable identifying arterial flow in the CCA using spectral Doppler. Residents reported an overall confidence level of 9 (SD + 1.1) on a scale of 1 to 10, with 1 indicating low confidence and 10 indicating high confidence, in identifying arterial flow in the CCA using spectral Doppler. Residents reported an overall confidence level of 8.75 (SD + 1.06) on a scale of 1 to 10, with 1 indicating low confidence and 10 indicating high confidence, in accurately identifying a pulse using carotid Doppler ultrasound on an actual patient receiving CPR after this training session. All residents agreed that carotid ultrasound can be used to accurately assess for a pulse, during the pulse check, of a patient receiving CPR. All residents agreed with the statement “Limited training in the use of spectral Doppler of the carotid artery is adequate to prepare for its use during CPR.” Following the training session, 32 (84%) residents felt that they would likely use this technique to check for pulses on a patient receiving CPR. Residents reported a likelihood of 8.3 (SD + 1.6) on a scale of 1 to 10, with 1 indicating low likelihood and 10 indicating high likelihood, of using this technique on a patient receiving CPR in the future. There was no incomplete data obtained from the questionnaires.

## Discussion

This study demonstrates the utility of a focused training session in successfully teaching EM residents how to visualize and perform spectral Doppler of the carotid artery. Several prior studies have demonstrated the utility of short training sessions in developing accurate diagnostic ability in emergency medicine (EM) physicians at multiple levels of previous experience [18,19]. For example, a similar study with a limited didactic and training session focused on the deep venous thrombosis-focused extended compression ultrasound application. Participants were able to successfully learn how to perform this study and took an average of 2.8 minutes to complete the full examination [20]. Another study that focused on training aimed at assessing the feasibility of teaching residents to perform common carotid artery velocity time interval (VTi) to assess volume responsiveness found that, on average, it took residents 2.9 minutes to perform the examination [21]. The efficient examination completion times in these studies demonstrate the feasibility and efficacy of incorporating focused, ultrasound training sessions into the resident curriculum and their role in ensuring resident competency in performing examinations in clinical settings.

In this study, nearly all participants were able to obtain optimal views of the CCA and appropriately utilize the spectral Doppler mode without any assistance. Additionally, the observer perceived that the participants were able to accomplish this with relatively low difficulty. The high overall success rate can be attributed to several factors. It may reflect the effectiveness of training. The success of instituting limited didactic sessions for POC ultrasound training has already been demonstrated among emergency residents. It was not unrealistic to predict that these groups would excel with similar training. The high success rates may also highlight the procedural competency of the residents involved, indicating that this is an ideal cohort to train and implement a protocol where CCA ultrasound is used during pulse checks in cardiac arrest.

While the time to accurately identify arterial flow in the carotid artery was longer than 10 seconds in many participants in this study, meeting the definition of prolonged pulse check, the advantage of CCA Doppler over manual palpation is not accounted for in this parameter. There was some variability and subjectivity when the expert physicians who were doing the evaluations marked the time. It is also likely that with slightly more training, their times would be faster.

Although the initial procurement of the CCA arterial flow signal is prolonged, the probe can be held over the CCA throughout resuscitation as it does not directly impede other interventions. Prior studies with porcine models have demonstrated the viability of using a prototypical multichannel continuous wave Doppler transducer array attached to the patient’s neck for continuous monitoring during resuscitation [22]. Such a device has the potential to enhance the efficiency of CPR by allowing the provider to tend to other resuscitative efforts rather than having to manually hold the ultrasound probe on the patient’s neck. Furthermore, CCA and femoral artery Doppler have been shown to be superior to manual palpation of pulses, offering a clear advantage once a signal has been established [23,24].

After training, participants reported significant gains in confidence levels in using spectral Doppler. Additionally, residents felt comfortable identifying arterial flow in the common carotid artery using spectral Doppler, and all participants agreed that limited training was adequate to prepare them for its use during CPR. However, participants acknowledged some challenges in applying this technique during CPR, rating it as moderately difficult. This indicates that while the technique is teachable and feasible, barriers such as time pressure, patient positioning, and CPR dynamics may require further exploration and training.

Limitations

This study has several limitations. First, it was conducted in a controlled environment using standardized patients, which may not fully replicate the challenges of obtaining Doppler waveforms during actual CPR. As this study was not done during active cardiopulmonary resuscitation, it does not account for added psychological stress and logistical complications. The use of standardized patients also does not account for expected variations in body habitus and anatomy that could make the examination more technically challenging in the live setting. Second, the sample size was relatively small and consisted of residents from two academic medical centers, limiting the generalizability of the findings. Performing a sample size calculation could have informed a decision to recruit more participants. The small sample size also limited us in performing further inferential statistics, as any subgroup would likely be too small to reliably capture meaningful differences. This study could also be improved upon by strengthening the questionnaire portion. It may have been beneficial to pilot the questionnaire prior to implementing the study. Exploring the use of other validated questionnaires used for similar training could have been potentially useful.

Most importantly, the study did not evaluate the impact of this technique on clinical outcomes, such as time to pulse detection or accuracy compared to manual palpation. Further research is needed to evaluate the use of carotid spectral Doppler in actual resuscitation scenarios. Future studies should focus on the impact of this technique on CPR efficiency, accuracy of pulse detection, and overall patient outcomes. The addition of a control group or a comparative modality for detecting pulses, which was absent in this study, could provide further data to argue for the use of carotid spectral Doppler. Additionally, exploring strategies to overcome perceived challenges, such as workflow integration and operator ergonomics, will be critical to its widespread adoption.

## Conclusions

This study demonstrates that emergency medicine residents can effectively learn and apply spectral Doppler to assess carotid pulses with minimal training. The findings support testing the inclusion of this technique as a method for pulse detection in live settings. Spectral Doppler of the CCA may be a reasonable alternative for identifying ROSC during pulse check of patients receiving CPR, but further research is warranted to evaluate its clinical impact and optimize its implementation during CPR.
